# Generalization of Bandlimited Functions and Applications to Quantum Probability Distributions

**DOI:** 10.3390/e28020198

**Published:** 2026-02-10

**Authors:** Leon Cohen

**Affiliations:** Institute for Quantum Science and Engineering, Texas A&M University, College Station, TX 77840, USA; leon.cohen@hunter.cuny.edu

**Keywords:** bandlimited functions, transforms, inequalities, quantum mechanics, wave functions

## Abstract

Bandlimited functions are functions whose Fourier transform is confined to a finite band of frequencies. We generalize this concept to representations other than the Fourier transform and show that this leads to a variety of inequalities in arbitrary representations. Several special cases are considered, including frequency, dilation, and the chirplet transform, among others. Examples are given to illustrate each result. We apply the results to quantum mechanical wave functions and probability distributions. For bounded momentum wave functions, we obtain explicit bounds on the position wave function and its derivatives, as well as bounds on the position probability distribution. We also consider the dual problem in which the position wave function is bounded, as in the case of a particle in a box with an arbitrary wave function, and obtain bounds on the corresponding momentum wave function and momentum probability distribution. The case of wave functions that are sums of a finite number of energy eigenfunctions is also developed, and bounds on the associated probability distributions are obtained. A number of specific examples are considered, including a truncated Gaussian wave function and a quantum bump wave function.

## 1. Introduction

Functions that are bandlimited in frequency have played a fundamental role in signal analysis and various aspects of physics and mathematics. By a bandlimited function, one means that its Fourier spectrum is limited to a band of values. In particular, for a function f(x) with Fourier transform F(k),

(1) $$F\left(k\right)=\frac{1}{\sqrt{2\pi }}\int_{-\infty }^{\infty }f\left(x\right)e^{-ikx}dx,$$

the function f(x) is said to be bandlimited if [1,2,3,4]

(2) $$F\left(k\right)=0\;\mathrm{for}\;\left|k\right|>\sigma ,$$

in which case, f(x) is given by

(3) $$f\left(x\right)=\frac{1}{\sqrt{2\pi }}\int_{-\sigma }^{\sigma }F\left(k\right)e^{ikx}dk.$$

Bandlimited signals are fundamental in signal processing for numerous reasons. They play a central role in the sampling of the corresponding time function, since one can choose a sampling rate that avoids aliasing and thereby allows exact reconstruction of the time function. They are also central to information transmission and channel capacity. Moreover, they provide an appropriate idealization of real physical signals, since signals encountered in practical systems, such as communication, radar, and sonar, are inherently bandwidth-limited.

Our aim is to generalize the concept of bandlimited functions to representations other than Fourier. In particular, for a self-adjoint operator A, we write

(4) $$A\;u_{\lambda }\left(x\right)\;=\;\lambda \;u_{\lambda }\left(x\right),$$

where λ and $u_{\lambda }\left(x\right)$ are the eigenvalues and eigenfunctions, respectively. (We consider the continuous case first.) Since A is self-adjoint, the eigenvalues are real and the eigenfunctions are complete and orthogonal. Any function, f(x), can be expanded as

(5) $$f\left(x\right)\;=\;\int_{-\infty }^{\infty }F\left(\lambda \right)u_{\lambda }\left(x\right)d\lambda$$

with

(6) $$F\left(\lambda \right)\;=\int_{-\infty }^{\infty }f\left(x\right)u_{\lambda }^{*}\left(x\right)dx.$$

F(λ) is called the transform of f(x) in the basis set $u_{\lambda }\left(x\right)$ [4,5,6,7]. We shall say that the function is bandlimited in the λ domain if, in that domain,

(7) $$F\left(\lambda \right)=0\;\mathrm{for}\;\left|\lambda \right|>\sigma ,$$

in which case,

(8) $$f\left(x\right)\;=\;\int_{-\sigma }^{\sigma }F\left(\lambda \right)u_{\lambda }\left(x\right)d\lambda .$$

We take the normalization factor to be

(9) $$N=\int_{-\sigma }^{\sigma }{\left|F(\lambda )\right|}^{2}d\lambda =\int_{-\infty }^{\infty }{\left|f(x)\right|}^{2}dx.$$

In the next section, we present the generalization and then discuss a number of special cases. We subsequently apply the results to the quantum mechanical case, where we obtain bounds on the probability distributions. We consider bandlimited momentum and position wave functions. The discrete case is then treated in general and also applied to the quantum mechanical case of expansions in terms of energy eigenfunctions. In addition, for some of the quantum examples, we consider bump functions, which are functions with compact support that are infinitely differentiable.

### Notation

For the generic transform variable, as indicated above, we use λ, and we denote the size of the finite band by 2σ. When we specialize to particular cases, however, we use the standard notation appropriate to each case. For example, in the case of spatial frequency, we use *k* in place of λ. In the quantum mechanical cases, we use the conventional notation, such as *p* for momentum. In addition, for the quantum case, we use *L* to denote the size of the band; that is, we take σ=L/2.

## 2. Generalization

We first summarize the main result and give the proof in Section 2.2.

### 2.1. Summary of Results

For a given bandlimited function F(λ), define the function g(x),

(10) $$g\left(x\right)=\int_{-\sigma }^{\sigma }F\left(\lambda \right)H\left(\lambda \right)u_{\lambda }\left(x\right)d\lambda ,$$

where H(λ) is an arbitrary *real* function (which is not, in general, bandlimited). Then

(11) $${\left|g(x)\right|}^{2}\leq N\int_{-\sigma }^{\sigma }{\left|H\left(\lambda \right)u_{\lambda }\left(x\right)\right|}^{2}d\lambda .$$

Different cases are obtained by choosing different representations, as exemplified by $u_{\lambda }\left(x\right)$, and also by choosing different H(λ).

Explicitly,

(12) $${\left|\int_{-\sigma }^{\sigma }F\left(\lambda \right)H\left(\lambda \right)u_{\lambda }\left(x\right)d\lambda \right|}^{2}\leq N\int_{-\sigma }^{\sigma }{\left|H\left(\lambda \right)u_{\lambda }\left(x\right)\right|}^{2}d\lambda .$$

In addition, we define

(13) g(x)=H(A)f(x),

where H(A) is the operator obtained by substituting A for λ in the function H(λ). If H(λ) is real, then H(A) is Hermitian. Equation (11) may hence be written as

(14) $${\left|H(A)f(x)\right|}^{2}\leq N\int_{-\sigma }^{\sigma }{\left|H\left(\lambda \right)u_{\lambda }\left(x\right)\right|}^{2}d\lambda ,$$

where

(15) $$f\left(x\right)\;=\;\int_{-\sigma }^{\sigma }F\left(\lambda \right)u_{\lambda }\left(x\right)d\lambda .$$

### 2.2. Proof

Taking the absolute square of both sides of Equation (10), we have [2]

(16) $${\left|g(x)\right|}^{2}={\left|\int_{-\sigma }^{\sigma }F\left(\lambda \right)H\left(\lambda \right)u_{\lambda }\left(x\right)d\lambda \right|}^{2}.$$

Using the Schwarz inequality in the form

(17) $${\left|\int_{-\sigma }^{\sigma }f_{1}^{*}\left(\lambda \right)\;f_{2}\left(\lambda \right)\;d\lambda \right|}^{2}\leq \left(\int_{-\sigma }^{\sigma }{\left|f_{1}\left(\lambda \right)\right|}^{2}\;d\lambda \right)\left(\int_{-\sigma }^{\sigma }{\left|f_{2}\left(\lambda \right)\right|}^{2}\;d\lambda \right),$$

with

(18) $$\begin{array}{cc} f_{1}\left(\lambda \right) & =F(\lambda ) \end{array}$$

(19) $$\begin{array}{cc} f_{2}\left(\lambda \right) & =H\left(\lambda \right)u_{\lambda }^{*}\left(x\right) \end{array}$$

and noting that H(λ) is real, we obtain

(20) $${\left|\int_{-\sigma }^{\sigma }F\left(\lambda \right)H\left(\lambda \right)u_{\lambda }\left(x\right)d\lambda \right|}^{2}\leq \int_{-\sigma }^{\sigma }{\left|F(\lambda )\right|}^{2}d\lambda \times \int_{-\sigma }^{\sigma }{\left|H\left(\lambda \right)u_{\lambda }\left(x\right)\right|}^{2}d\lambda .$$

Imposing Equation (9), we have

(21) $${\left|g(x)\right|}^{2}\leq N\int_{-\sigma }^{\sigma }{\left|H\left(\lambda \right)u_{\lambda }\left(x\right)\right|}^{2}d\lambda .$$

Explicitly,

(22) $${\left|\int_{-\sigma }^{\sigma }F\left(\lambda \right)H\left(\lambda \right)u_{\lambda }\left(x\right)d\lambda \right|}^{2}\leq N\int_{-\sigma }^{\sigma }{\left|H\left(\lambda \right)u_{\lambda }\left(x\right)\right|}^{2}d\lambda .$$

Equality is achieved for

(23) $$F\left(\lambda \right)=\sqrt{N}H\left(\lambda \right)u_{\lambda }^{*}\left(x\right)\;\mathrm{for}-\sigma <\lambda <\sigma .$$

**Operator form.** From Equation (4), it follows that [5,6,7,8]

(24) $$H\left(A\right)u_{\lambda }\left(x\right)=H\left(\lambda \right)u_{\lambda }\left(x\right),$$

and therefore,

(25) $$\begin{array}{cc} g(x) & =\int_{-\sigma }^{\sigma }F\left(\lambda \right)H\left(A\right)u_{\lambda }\left(x\right)d\lambda \end{array}$$

(26) $$\begin{array}{cc} & =H\left(A\right)\int_{-\sigma }^{\sigma }F\left(\lambda \right)u_{\lambda }\left(x\right)d\lambda , \end{array}$$

which shows that g(x) may be obtained by way of

(27) g(x)=H(A)f(x),

in which case, Equation (22) may be written as

(28) $${\left|H(A)f(x)\right|}^{2}\leq N\int_{-\sigma }^{\sigma }{\left|H\left(\lambda \right)u_{\lambda }\left(x\right)\right|}^{2}d\lambda .$$

## 3. Special Cases of H(λ)

Since H(λ) is arbitrary, by taking different functions for H(λ), one obtains different cases and different inequalities.

### 3.1. H(λ)=1

For H(λ)=1, we have from Equation (14)

(29) g(x)=f(x),

and therefore, Equation (11) gives

(30) $${\left|f(x)\right|}^{2}\leq N\int_{-\sigma }^{\sigma }{\left|u_{\lambda }\left(x\right)\right|}^{2}d\lambda .$$

### 3.2. H(λ)=λ

Taking

(31) H(λ)=λ,

which is equivalent to taking

(32) g(x)=Af(x),

Equation (11) gives

(33) $${\left|Af(x)\right|}^{2}\leq N\int_{-\sigma }^{\sigma }{\left|\lambda u_{\lambda }\left(x\right)\right|}^{2}d\lambda$$

or

(34) $${\left|g(x)\right|}^{2}\leq N\int_{-\sigma }^{\sigma }{\left|\lambda u_{\lambda }\left(x\right)\right|}^{2}d\lambda .$$

### 3.3. $H\left(\lambda \right)=\lambda^{n}$

For $H\left(\lambda \right)=\lambda^{n}$,

(35) $$g\left(x\right)=A^{n}f\left(x\right),$$

we have

(36) $${\left|A^{n}f\left(x\right)\right|}^{2}\leq N\int_{-\sigma }^{\sigma }{\left|\lambda^{n}u_{\lambda }\left(x\right)\right|}^{2}d\lambda$$

or

(37) $${\left|g(x)\right|}^{2}\leq N\int_{-\sigma }^{\sigma }{\left|\lambda^{n}u_{\lambda }\left(x\right)\right|}^{2}d\lambda .$$

## 4. Frequency

The spatial frequency operator, A, is

(38) $$A=\frac{1}{i}\frac{d}{dx}.$$

This example is the standard case of bandlimited functions, but we use the method presented above to illustrate the general procedure.

For spatial frequency, we use the common notation, *k*; that is, we take λ=k. The eigenvalue problem is

(39) $$\frac{1}{i}\frac{d}{dx}u_{k}\left(x\right)=ku_{k}\left(x\right),$$

and the eigenfunctions normalized to a delta function are

(40) $$u_{k}\left(x\right)=\frac{1}{\sqrt{2\pi }}\;e^{ikx}.$$

Now,

(41) $$f\left(x\right)\;=\;\int_{-\sigma }^{\sigma }F\left(k\right)u_{k}\left(x\right)\;dk$$

and applying Equation (30), we have that

(42) $${\left|f(x)\right|}^{2}\leq N\int_{-\sigma }^{\sigma }{\left|u_{k}\left(x\right)\right|}^{2}d\lambda =\frac{N}{2\pi }\int_{-\sigma }^{\sigma }d\lambda .$$

The integral gives 2σ and therefore [2]

(43) $${\left|f(x)\right|}^{2}\leq \frac{N\sigma }{\pi }$$

or

(44) $$\left|f(x)\right|\leq \sqrt{\frac{N\sigma }{\pi }}.$$

For

(45) $$g\left(x\right)=Af\left(x\right)=\frac{1}{i}\frac{d}{dx}f\left(x\right),$$

Equation (33) gives

(46) $${\left|\frac{1}{i}\frac{d}{dx}f\left(x\right)\right|}^{2}\leq N\int_{-\sigma }^{\sigma }{\left|\lambda \frac{1}{\sqrt{2\pi }}e^{i\lambda x}\right|}^{2}d\lambda ,$$

and therefore,

(47) $$\left|\frac{d}{dx}f\left(x\right)\right|\leq \sqrt{\frac{N\sigma^{3}}{3\pi }}.$$

For the *n*th power case where

(48) $$g\left(x\right)=A^{n}f\left(x\right)={\left(\frac{1}{i}\frac{d}{dx}\right)}^{n}f\left(x\right),$$

Equation (36) gives

(49) $${\left|{\left(\frac{1}{i}\frac{d}{dx}\right)}^{n}f\left(x\right)\right|}^{2}\leq N\int_{-\sigma }^{\sigma }{\left|\lambda^{n}\frac{1}{\sqrt{2\pi }}e^{i\lambda x}\right|}^{2}d\lambda ,$$

which simplifies to

(50) $$\left|\frac{d^{n}}{dx^{n}}f\left(x\right)\right|\leq \sqrt{\frac{N}{\pi }\frac{\sigma^{2n+1}}{2n+1}}.$$

**Amplitude and phase constraints**. It is of interest to write the function f(x) in terms of its amplitude and phase

(51) $$f\left(x\right)=A\left(x\right)e^{i\varphi (x)},$$

in which case,

(52) $$\frac{d}{dx}f\left(x\right)=\left(\frac{dA}{dx}+iA\frac{d\varphi }{dx}\right)e^{i\varphi (x)}$$

and

(53) $${\left|\frac{d}{dx}f\left(x\right)\right|}^{2}={\left(\frac{dA}{dx}\right)}^{2}+{\left(A\frac{d\varphi }{dx}\right)}^{2}.$$

Equations (44) and (47) give

(54) $${\left|A(x)\right|}^{2}\leq \frac{N\sigma }{\pi }$$

and

(55) $${\left(\frac{dA}{dx}\right)}^{2}+{\left(A\omega_{i}\right)}^{2}\leq \frac{N\sigma^{3}}{3\pi },$$

where

(56) $$\omega_{i}=\frac{d\varphi }{dx},$$

which is the instantaneous spatial frequency [7,8,9,10,11]. Rewriting Equation (55) as

(57) $$\omega_{i}^{2}\leq \frac{1}{A^{2}}\left(\frac{N\sigma^{3}}{3\pi }-{\left(\frac{dA}{dx}\right)}^{2}\right)$$

shows how the instantaneous spatial frequency is constrained.

### 4.1. Example

Consider the spatial spectrum given by

(58) $$F\left(k\right)=\sqrt{\frac{15}{16\sigma }}\left[1-{\left(\frac{k}{\sigma }\right)}^{2}\right]\;\mathrm{for}\;\left|k\right|\leq \sigma .$$

The function is normalized to one

(59) $$N=\int_{-\sigma }^{\sigma }{\left|F(k)\right|}^{2}dk=1.$$

The spatial function is

(60) $$f\left(x\right)=\frac{1}{\sqrt{2\pi }}\int_{-\sigma }^{\sigma }F\left(k\right)e^{ikx}dk.$$

Using Equation (44) with N=1, we have

(61) $$\left|f(x)\right|\leq \sqrt{\frac{\sigma }{\pi }}.$$

For this case, the spatial function can be calculated exactly

(62) $$f\left(x\right)=\sqrt{\frac{15}{2\pi }}\frac{1}{\sigma^{5/2}}\frac{1}{x^{3}}\left[\sin(\sigma x)-\sigma x\cos\sigma x\right],$$

and therefore, we have

(63) $$\left|\sqrt{\frac{15}{2\pi }}\frac{1}{\sigma^{5/2}}\frac{1}{x^{3}}\left[\sin(\sigma x)-\sigma x\cos\sigma x\right]\right|\leq \sqrt{\frac{\sigma }{\pi }}.$$

### 4.2. Example: $H\left(k\right)=e^{-ak}$

Consider the case

(64) $$H\left(k\right)=e^{-ak}$$

with *a* real

(65) $$H\left(A\right)=e^{ia\frac{d}{dx}}.$$

We have [8,12]

(66) $$\begin{array}{cc} g(x) & =H(A)f(x) \end{array}$$

(67) $$\begin{array}{cc} & =e^{ia\frac{d}{dx}}f\left(x\right)=f\left(x+ia\right). \end{array}$$

Using Equation (14), we obtain that

(68) $${\left|f(x+ia)\right|}^{2}\leq N\int_{-\sigma }^{\sigma }{\left|H\left(k\right)u_{k}\left(x\right)\right|}^{2}dk$$

or

(69) $${\left|f(x+ia)\right|}^{2}\leq N\int_{-\sigma }^{\sigma }{\left|e^{-ak}\right|}^{2}dk,$$

which evaluates to

(70) $${\left|f(x+ia)\right|}^{2}\leq \frac{N}{a}\sinh\left(2a\sigma \right).$$

Notice that the left-hand side depends on *x* but the right-hand side does not. This inequality holds as long as f(x) is a bandlimited function.

### 4.3. Example: Raised Cosine

Consider the normalized raised cosine function in *k* space,

(71) $$F\left(k\right)=\;\sqrt{\frac{1}{3\sigma }}\left(1+\cos\frac{\pi k}{\sigma }\right)\;\mathrm{for}\;\left|k\right|\leq \sigma .$$

The spatial function is

(72) $$f\left(x\right)=\frac{1}{\sqrt{2\pi }}\int_{-\sigma }^{\sigma }F\left(k\right)e^{ikx}dk$$

and, per Equations (47) and (50), satisfies

(73) $$\left|f(x)\right|\leq \sqrt{\frac{2\sigma }{\pi }}$$

and

(74) $$\left|\frac{d^{n}}{dx^{n}}f\left(x\right)\right|\leq \sqrt{\frac{1}{\pi }\frac{2\sigma^{2n+1}}{2n+1}}.$$

For this case, f(x) can be calculated exactly

(75) $$f\left(x\right)=-\frac{\pi }{3\sigma }\frac{\sin\sigma x}{x(\sigma^{2}x^{2}-\pi^{2})},$$

and therefore,

(76) $$\left|\frac{\pi }{3\sigma }\frac{\sin\sigma x}{x(\sigma^{2}x^{2}-\pi^{2})}\right|\leq \sqrt{\frac{2\sigma }{\pi }},$$

and for the first derivative, we have

(77) $$\left|\frac{d}{dx}\frac{\pi }{3\sigma }\frac{\sin\sigma x}{x(\sigma^{2}x^{2}-\pi^{2})}\right|\leq \sqrt{\frac{2\sigma^{3}}{3\pi }}.$$

## 5. Quantum Mechanical Case

We specialize to the quantum mechanical case where the momentum wave function φ(p) is given by [5,6]

(78) $$\varphi \left(p\right)=\frac{1}{\sqrt{2\pi \hbar }}\int_{-\infty }^{\infty }\psi \left(x\right)e^{-ipx/\hbar }dx.$$

Inversely, the position wave function is

(79) $$\psi \left(x\right)=\frac{1}{\sqrt{2\pi \hbar }}\int_{-\infty }^{\infty }\varphi \left(p\right)e^{ipx/\hbar }dp.$$

One can transform the results of the previous section using k=p/ℏ; however, it is instructive to use the momentum operator

(80) $$p=\frac{\hbar }{i}\frac{d}{dx}$$

and rederive the results. The eigenvalue problem for momentum is

(81) $$\frac{\hbar }{i}\frac{d}{dx}u_{p}\left(x\right)=p\;u_{p}\left(x\right),$$

which gives the eigenfunctions

(82) $$u_{p}\left(x\right)=\frac{1}{\sqrt{2\pi \hbar }}\;e^{ipx/\hbar }.$$

We consider bandlimited momentum wave functions where for

(83) $$\varphi \left(p\right)=0\;\mathrm{for}\;\left|p\right|>\frac{L}{2},$$

in which case,

(84) $$\psi \left(x\right)\;=\;\frac{1}{\sqrt{2\pi \hbar }}\int_{-L/2}^{L/2}\varphi \left(p\right)e^{ipx/\hbar }dp.$$

We take the normalization factor to be

(85) $$N=\int_{-L/2}^{L/2}{\left|\varphi (p)\right|}^{2}dp=\int_{-\infty }^{\infty }{\left|\psi (x)\right|}^{2}dx.$$

Consider the position wave function, g(x), corresponding to the momentum wave function of φ(p)H(p), where H(p) is an arbitrary real function

(86) $$g\left(x\right)=\frac{1}{\sqrt{2\pi \hbar }}\int_{-L/2}^{L/2}\varphi \left(p\right)H\left(p\right)e^{ipx/\hbar }dp.$$

Using the Schwarz inequality in the form

(87) $${\left|\int_{-L/2}^{L/2}f_{1}^{*}\left(p\right)\;f_{2}\left(p\right)\;dp\right|}^{2}\leq \left(\int_{-L/2}^{L/2}{\left|f_{1}\left(p\right)\right|}^{2}\;dp\right)\left(\int_{-L/2}^{L/2}{\left|f_{2}\left(p\right)\right|}^{2}\;dp\right),$$

with

(88) $$\begin{array}{cc} f_{1}\left(\lambda \right) & =\varphi (p) \end{array}$$

(89) $$\begin{array}{cc} f_{2}\left(\lambda \right) & =H\left(p\right)\frac{1}{\sqrt{2\pi \hbar }}e^{-ipx/\hbar }, \end{array}$$

we obtain

(90) $${\left|\int_{-L/2}^{L/2}\varphi \left(p\right)H\left(p\right)\frac{1}{\sqrt{2\pi \hbar }}e^{ipx/\hbar }dp\right|}^{2}\leq \int_{-L/2}^{L/2}{\left|\varphi (p)\right|}^{2}dp\times \int_{-L/2}^{L/2}{\left|H\left(p\right)\frac{1}{\sqrt{2\pi \hbar }}e^{ipx/\hbar }\right|}^{2}dp.$$

For the case

(91) $$H\left(p\right)=p^{n}$$

and noting that

(92) $$\int_{-L/2}^{L/2}\varphi \left(p\right)H\left(p\right)\frac{1}{\sqrt{2\pi \hbar }}e^{ipx/\hbar }dp={\left(\frac{\hbar }{i}\right)}^{n}\frac{d^{n}}{dx^{n}}\psi \left(x\right),$$

we obtain

(93) $${\left|{\left(\frac{\hbar }{i}\right)}^{n}\frac{d^{n}}{dx^{n}}\psi \left(x\right)\right|}^{2}\leq N\int_{-L/2}^{L/2}{\left|p^{n}\frac{1}{\sqrt{2\pi \hbar }}e^{ipx/\hbar }\right|}^{2}dp$$

or

(94) $${\left|\frac{d^{n}}{dx^{n}}\psi \left(x\right)\right|}^{2}\leq \frac{N}{\pi (2n+1)}{\left(\frac{L}{2\hbar }\right)}^{2n+1}.$$

For the n=0 case,

(95) $${\left|\psi (x)\right|}^{2}\leq \frac{NL}{2\hbar \pi }.$$

That is,

(96) $$\mathrm{Probability}\;\mathrm{of}\;\mathrm{position}={\left|\psi (x)\right|}^{2}\;\leq \frac{NL}{2\hbar \pi },$$

and for the derivative of the wave function, using Equation (95), we have

(97) $${\left|\frac{d}{dx}\psi \left(x\right)\right|}^{2}\leq \frac{N}{24\pi }{\left(\frac{L}{\hbar }\right)}^{3}.$$

What this shows is that if the momentum wave function is bandlimited, then the magnitude of the position wave function is bounded. Moreover, the oscillations of the position wave function, as exemplified by the derivative of the wave function, is also limited in magnitude.

### 5.1. Example: Constant φ(p) Case

For the normalized constant momentum wave function in the range −L/2 to L/2,

(98) $$\varphi \left(p\right)=\sqrt{\frac{1}{L}}\;\left|p\right|\leq L/2.$$

Equation (95) gives that

(99) $$\mathrm{Probability}\;\mathrm{of}\;\mathrm{position}\;={\left|\psi (x)\right|}^{2}\leq \frac{L}{2\hbar \pi }.$$

The position wave function can be calculated exactly,

(100) $$\psi \left(x\right)=\frac{1}{\sqrt{2\pi \hbar }}\sqrt{\frac{1}{L}}\int_{-L/2}^{L/2}e^{ipx/\hbar }dp=\sqrt{\frac{2\hbar }{\pi L}}\frac{\sin Lx/2\hbar }{x}.$$

Equation (99) gives

(101) $$\mathrm{Probability}\;\mathrm{of}\;\mathrm{position}\;=\left|\sqrt{\frac{2\hbar }{\pi L}}\frac{\sin Lx/2\hbar }{x}\right|\leq \frac{L}{2\hbar \pi }.$$

We also have, from Equation (94), that

$${\left|{\left(\frac{\hbar }{i}\right)}^{n}\frac{d^{n}}{dx^{n}}\psi \left(x\right)\right|}^{2}\leq \int_{-L/2}^{L/2}{\left|p^{n}\frac{1}{\sqrt{2\pi \hbar }}e^{ipx/\hbar }\right|}^{2}dp$$

or

(102) $${\left|\frac{d^{n}}{dx^{n}}\sqrt{\frac{2\hbar }{L\pi }}\frac{\sin Lx/2\hbar }{x}\right|}^{2}\leq \frac{1}{\pi (2n+1)}{\left(\frac{L}{2\hbar }\right)}^{2n+1}.$$

### 5.2. Example: Truncated Momentum Gaussian Wave Function

We consider the truncated and normalized Gaussian wave function

(103) $$\varphi \left(p\right)=C\;\exp\;\left(-\frac{p^{2}}{L^{2}}-ix_{0}p/\hbar \right)\;\left|p\right|\leq L/2,$$

where

(104) $$C=\frac{1}{\sqrt{L\mathrm{erf}(1/\sqrt{2})}}\frac{1}{{\left(\pi /2\right)}^{1/4}}.\;$$

Using Equation (95), we have that the probability of position is bounded

(105) $$\mathrm{Probability}\;\mathrm{of}\;\mathrm{position}={\left|\psi (x)\right|}^{2}\leq \frac{L}{2\hbar \pi },$$

and also all the derivatives of the wave function are bounded by

(106) $${\left|\frac{d^{n}}{dx^{n}}\psi \left(x\right)\right|}^{2}\leq \frac{1}{\pi (2n+1)}{\left(\frac{L}{2\hbar }\right)}^{2n+1}.$$

For this case, the position wave function is calculated to be

(107) ψ(x)=L/ℏerf(1/2)1(2π)1/4e−L2(x−x0)24ℏ2Reerf12+i(x−x0)L2ℏ,

and therefore,

(108) Probability of position=L/ℏerf(1/2)12π)e−L2(x−x0)22ℏ2Reerf12+i(x−x0)L2ℏ2≤L2ℏπ.

We emphasize that one does not have to calculate ψ(x) to know the bounds on it.

### 5.3. Example: Quantum Raised Cosine in Momentum Space

For the normalized momentum wave function,

(109) $$\varphi \left(p\right)=\;\sqrt{\frac{2}{3L}}\left(1+\cos\;\frac{2\pi p}{L}\right)\;\mathrm{for}\;\left|p\right|\leq L/2.$$

That is the probability of position is bounded by

(110) $$\mathrm{Probability}\;\mathrm{of}\;\mathrm{position}\;={\left|\psi (x)\right|}^{2}\leq \frac{L}{2\hbar \pi },$$

and the derivatives satisfy

(111) $$\left|\frac{d^{n}}{dx^{n}}\psi \left(x\right)\right|\leq \frac{1}{\pi (2n+1)}{\left(\frac{L}{2\hbar }\right)}^{2n+1}.$$

The spatial function can be calculated exactly

(112) $$\psi \left(x\right)=-\frac{8\pi^{2}}{\sqrt{3\pi }}\;\sqrt{\frac{L}{\hbar }}\;\frac{\sin\left(\frac{Lx}{2\hbar }\right)}{\left(\frac{Lx}{\hbar }\right)\left[{\left(\frac{Lx}{\hbar }\right)}^{2}-4\pi^{2}\right]},$$

and hence, the probability distribution is given by

(113) $${\left|\psi \left(x\right)\right|}^{2}=\frac{64\pi^{3}}{3}\;\frac{L}{\hbar }\;\frac{\sin^{2}\left(\frac{Lx}{2\hbar }\right)}{{\left(\frac{Lx}{\hbar }\right)}^{2}{\left[{\left(\frac{Lx}{\hbar }\right)}^{2}-4\pi^{2}\right]}^{2}}.$$

Therefore, we may write Equation (110)

(114) $$\mathrm{Probability}\;\mathrm{of}\;\mathrm{position}\;=\frac{64\pi^{3}}{3}\;\frac{L}{\hbar }\;\frac{\sin^{2}\left(\frac{Lx}{2\hbar }\right)}{{\left(\frac{Lx}{\hbar }\right)}^{2}{\left[{\left(\frac{Lx}{\hbar }\right)}^{2}-4\pi^{2}\right]}^{2}}\leq \frac{L}{2\hbar \pi }.$$

We note that, in the limit x→0,

(115) $$\psi \left(0\right)=\sqrt{\frac{L}{3\pi \hbar }},$$

and for the singularities at x=±2πℏ/L, we have

(116) $$\psi \;\left(\pm \frac{2\pi \hbar }{L}\right)=\frac{1}{2}\sqrt{\frac{L}{3\pi \hbar }}.$$

## 6. Scale/Dilation

The scale or dilatation operator, C, is defined by [13,14]

(117) $$C\;=\frac{1}{2i}\;\left(x\frac{d}{dx}+\frac{d}{dx}x\right),\;$$

which may also be written as

(118) $$C\;=\;\frac{1}{i}\left(x\frac{d}{dx}+\frac{1}{2}\right)=\frac{1}{i}\left(\frac{d}{dx}x-\frac{1}{2}\right).$$

The eigenvalue problem

(119) $$C\;u_{\lambda }\left(x\right)=\lambda u_{\lambda }\left(x\right)\;\;$$

gives the eigenfunctions [13]

(120) $$u_{\lambda }\left(x\right)\;=\;\;\frac{1}{\sqrt{2\pi }}\;\frac{\;\;e^{i\lambda \ln x}}{\sqrt{x}}\;,\;x\;\geq 0.$$

They are normalized so that

(121) $$\int_{0}^{\infty }u_{\lambda }^{*}\left(x\right)u_{\lambda^{\prime }}\left(x\right)\;dx=\delta \left(\lambda -\lambda^{\prime }\right)$$

(122) $$\int_{-\infty }^{\infty }u^{*}\left(\lambda ,x^{\prime }\right)u_{\lambda }\left(x\right)d\lambda =\delta \left(x-x^{\prime }\right)\;\;\;\;\;\;\;\;\;x,x^{\prime }\geq 0.\;$$

Any function defined on the positive *x* axis may be expanded as

(123) $$f\left(x\right)=\frac{1}{\sqrt{2\pi }}\;\int_{-\infty }^{\infty }D\left(\lambda \right)\frac{e^{i\lambda \ln x}}{\sqrt{x}}d\lambda \;\;\;\;\;\;;\;\;\;\;\;x\geq 0,$$

where the scale transform, D(λ), is given by

(124) $$D\left(\lambda \right)=\frac{1}{\sqrt{2\pi }}\int_{0}^{\infty }f\left(x\right)\frac{e^{-i\lambda \ln x}}{\sqrt{x}}dx.\;$$

A fundamental property of the scale operator is

(125) $$\;\;e^{i\ln\theta C}\;f\left(x\right)\;=\;\sqrt{\theta }\;f\left(\theta x\right).$$

For the bandlimited case where

(126) $$D\left(\lambda \right)=0\;\mathrm{for}\;\left|\lambda \right|>\sigma ,$$

Equation (14) gives

(127) $${\left|H(C)f(x)\right|}^{2}\leq N\int_{-\sigma }^{\sigma }{\left|H\left(\lambda \right)\frac{1}{\sqrt{2\pi }}\frac{e^{i\lambda \ln x}}{\sqrt{x}}\right|}^{2}d\lambda .$$

For

(128) $$H\left(\lambda \right)=\lambda^{n},$$

we obtain that

(129) $${\left|H(C)f(x)\right|}^{2}\leq \frac{N}{\pi x}\frac{\sigma^{2n+1}}{2n+1}$$

or explicitly

(130) $${\left|{\left[\frac{1}{2i}\;\left(x\frac{d}{dx}+\frac{d}{dx}x\right)\right]}^{n}f\left(x\right)\right|}^{2}\leq \frac{N}{\pi x}\frac{\sigma^{2n+1}}{2n+1}.$$

Equivalently,

(131) $$\frac{1}{2^{n}}\left|\;{\left(x\frac{d}{dx}+\frac{d}{dx}x\right)}^{n}f\left(x\right)\right|\leq \sqrt{\frac{N}{\pi x}\frac{\sigma^{2n+1}}{2n+1}}.$$

For the n=0 case,

(132) $$\left|f(x)\right|\leq \sqrt{\frac{N\sigma }{\pi x}},$$

and for n=1, we have

(133) $$\left|Cf(x)\right|\leq \sqrt{\frac{N\sigma^{3}}{3\pi x}}.$$

Explicitly,

(134) $$\left|\frac{1}{2}\;\left(x\frac{d}{dx}+\frac{d}{dx}x\right)f\left(x\right)\right|\leq \sqrt{\frac{N\sigma^{3}}{3\pi x}}.$$

### Constant D(λ) Case

For the case where

(135) D(λ)=1,

Equation (123) gives that

(136) $$\begin{array}{cc} f(x) & =\frac{1}{\sqrt{2\pi }}\int_{-\sigma }^{\sigma }\frac{e^{i\lambda \ln x}}{\sqrt{x}}d\lambda \;\;\;\;\;\;\;\;\;\;\;x\geq 0 \end{array}$$

(137) $$\begin{array}{cc} & =\frac{1}{\sqrt{2\pi }}\frac{1}{\sqrt{x}}\frac{2}{\ln x}\sin\left(\sigma \ln x\right)\;\;\;\;\;\;;\;\;\;\;\;\;\;x\geq 0. \end{array}$$

Additionally,

(138) $$N=\int_{-\sigma }^{\sigma }{\left|D(\lambda )\right|}^{2}d\lambda =2\sigma .$$

Using Equation (132), we obtain

(139) $$\left|\frac{1}{\sqrt{2\pi }}\frac{1}{\sqrt{x}}\frac{2}{\ln x}\sin\left(\sigma \ln x\right)\right|\leq \sqrt{\frac{2\sigma^{2}}{\pi x}}.$$

## 7. $A=\alpha x-i\beta \frac{d}{\mathrm{dx}}$

Consider the operator

(140) $$A=\alpha x-i\beta \frac{d}{dx}$$

with real α and β. This operator and its associated eigenfunctions are related to coherent states in quantum mechanics [15,16,17] and to the chirplet transform in signal processing [18,19,20]. Solving the eigenvalue problem

(141) $$\left(\alpha x-i\beta \frac{d}{dx}\right)u_{\lambda }\left(x\right)=\lambda u_{\lambda }\left(x\right)$$

gives

(142) $$u_{\lambda }\left(x\right)=\frac{1}{\sqrt{2\pi \beta }}\;e^{i(\lambda x-\alpha x^{2}/2)/\beta },$$

where we have normalized to a delta function. Hence, we have the following transform pairs:

(143) $$F\left(\lambda \right)\;=\;\frac{1}{\sqrt{2\pi \beta }}\int_{-\infty }^{\infty }f\left(x\right)e^{-i(\lambda x-\alpha x^{2}/2)/\beta }dx$$

(144) $$f\left(x\right)\;=\;\frac{1}{\sqrt{2\pi \beta }}\int_{-\infty }^{\infty }F\left(\lambda \right)e^{i(\lambda x-\alpha x^{2}/2)/\beta }d\lambda .$$

For the bandlimited case where

(145) $$F\left(\lambda \right)=0\;\mathrm{for}\;\left|\lambda \right|>\sigma ,$$

the position function is

(146) $$f\left(x\right)\;=\;\frac{1}{\sqrt{2\pi \beta }}\int_{-\sigma }^{\sigma }F\left(\lambda \right)e^{i(\lambda x-\alpha x^{2}/2)/\beta }d\lambda .$$

Taking

(147) $$H\left(\lambda \right)=\lambda^{n}$$

in Equation (14) results in

(148) $${\left|{\left(\alpha x-i\beta \frac{d}{dx}\right)}^{n}f\left(x\right)\right|}^{2}\leq N\frac{1}{\pi \beta }\frac{\sigma^{2n+1}}{2n+1}$$

We list the first few cases of interest. For the for n=0 case,

(149) $${\left|f(x)\right|}^{2}\leq N\frac{\sigma }{\pi \beta }$$

and for n=1, we have

(150) $${\left|\left(\alpha x-i\beta \frac{d}{dx}\right)f\left(x\right)\right|}^{2}\leq N\frac{\sigma^{3}}{3\pi \beta }.$$

For n=2, we obtain

(151) $${\left|{\left(\alpha x-i\beta \frac{d}{dx}\right)}^{2}f\left(x\right)\right|}^{2}\leq N\frac{\sigma^{5}}{5\pi \beta }.$$

### Constant F(λ) Case

For F(λ)=1, we have

(152) $$f\left(x\right)\;=\;\frac{1}{\sqrt{2\pi \beta }}\int_{-\sigma }^{\sigma }e^{i(\lambda x-\alpha x^{2}/2)/\beta }d\lambda ,$$

which evaluates to

(153) $$f\left(x\right)\;=\frac{1}{x}\sqrt{\frac{2\beta }{\pi }}e^{-i\alpha x^{2}/2\beta }\sin\left(\sigma x/\beta \right).$$

Therefore, Equation (30) (with N=2σ) gives

(154) $${\left|\frac{1}{x}\sqrt{\frac{2\beta }{\pi }}e^{-i\alpha x^{2}/2\beta }\sin\left(\sigma x/\beta \right)\right|}^{2}\leq \frac{2\sigma^{2}}{\pi \beta }$$

or

(155) $${\left|\frac{1}{x}\sqrt{\frac{2\beta }{\pi }}\sin\left(\sigma x/\beta \right)\right|}^{2}\leq \frac{2\sigma^{2}}{\pi \beta }.$$

## 8. Fractional Fourier Transform

In standard notation, the fractional Fourier transform is defined by [21,22,23]

(156) $$g\left(u\right)=\int_{-\infty }^{\infty }K_{\theta }\left(u,x\right)f\left(x\right)\;dx,$$

and the inverse by

(157) $$f\left(x\right)=\int_{-\infty }^{\infty }K_{\theta }^{*}\left(u,x\right)g\left(u\right)\;du,$$

where

(158) $$K_{\theta }\left(u,x\right)=\sqrt{\frac{1-i\cot\theta }{2\pi }}\exp\;\left[\frac{i}{2}\left[(x^{2}+u^{2})\cot\theta -2xu\;\csc\theta \right]\right].$$

In this formulation, θ is a parameter, and for different values of θ, one gets different transforms. In particular, for example, for θ=π/2, one obtains the standard Fourier transform. The kernel of the transformation, $K_{\theta }\left(u,x\right),$ satisfies

(159) $$\begin{array}{cc} \int_{-\infty }^{\infty }K_{\theta }\left(u,x\right)K_{\theta }^{*}\left(u^{\prime },x\right)dx & =\delta (u-u^{\prime }) \end{array}$$

(160) $$\begin{array}{cc} \int_{-\infty }^{\infty }K_{\theta }\left(u,x\right)K_{\theta }^{*}\left(u,x^{\prime }\right)du & =\delta (x-x^{\prime }). \end{array}$$

$K_{\theta }\left(u,x\right)$ are the eigenfunctions of the operator A, where

(161) $$A=x\cos\theta +i\sin\theta \frac{d}{dx}.$$

That is,

(162) $$\left(x\cos\theta +i\sin\theta \frac{d}{dx}\right)K_{\theta }\left(u,x\right)=uK_{\theta }\left(u,x\right).$$

This form fits with minor modifications, the general scheme we developed in Section 2 and Section 3.

For a bandlimited g(u), we write

(163) $$f\left(x\right)=\int_{-\sigma }^{\sigma }K_{\theta }^{*}\left(u,x\right)g\left(u\right)\;du.$$

To put this formulation in our notation, we take u=λ

(164) $$\begin{array}{cc} u_{\lambda }\left(x\right) & =K_{\theta }^{*}\left(\lambda ,x\right) \end{array}$$

(165) $$\begin{array}{cc} F(\lambda ) & =g(u). \end{array}$$

We can then write Equation (22) as

(166) $${\left|\int_{-\sigma }^{\sigma }g\left(u\right)H\left(u\right)K_{\theta }^{*}\left(u,x\right)du\right|}^{2}\leq N\int_{-\sigma }^{\sigma }{\left|H\left(u\right)K_{\theta }^{*}\left(u,x\right)\right|}^{2}du.$$

In this paper, we just consider the cases where H(u)=1 and H(u)=u.

For H(u)=1, Equation (166) is

(167) $${\left|\int_{-\sigma }^{\sigma }g\left(u\right)K_{\theta }^{*}\left(u,x\right)du\right|}^{2}\leq N\int_{-\sigma }^{\sigma }{\left|K_{\theta }^{*}\left(u,x\right)\right|}^{2}du,$$

giving

(168) $${\left|f(x)\right|}^{2}\leq N\int_{-\sigma }^{\sigma }{\left|K_{\theta }^{*}\left(u,x\right)\right|}^{2}du$$

now

(169) $${\left|K_{\theta }^{*}\left(\lambda ,x\right)\right|}^{2}={\left|\frac{1-i\cot\theta }{2\pi }\right|}^{2}=\frac{1}{2\pi \left|\sin\theta \right|},$$

and furthermore,

(170) $$\int_{-\sigma }^{\sigma }{\left|K_{\theta }^{*}\left(\lambda ,x\right)\right|}^{2}d\lambda =\frac{\sigma }{\pi \left|\sin\theta \right|}.$$

Equation (168) then gives

(171) $${\left|f(x)\right|}^{2}\leq N\frac{\sigma }{\pi \left|\sin\theta \right|}.$$

For the case H(u)=u, Equation (166) is

(172) $${\left|\int_{-\sigma }^{\sigma }g\left(u\right)uK_{\theta }^{*}\left(u,x\right)du\right|}^{2}\leq N\int_{-\sigma }^{\sigma }{\left|uK_{\theta }^{*}\left(u,x\right)\right|}^{2}du.$$

Consider

(173) $$\begin{array}{cc} \left|\int_{-\sigma }^{\sigma }g\left(u\right)uK_{\theta }^{*}\left(u,x\right)du\right| & =\left|\int_{-\sigma }^{\sigma }g\left(u\right)\left(\cos\theta +i\sin\theta \frac{d}{dx}\right)K_{\theta }\left(u,x\right)du\right| \end{array}$$

(174) $$\begin{array}{cc} & =\left(\cos\theta +i\sin\theta \frac{d}{dx}\right)f\left(x\right), \end{array}$$

and therefore, we have

(175) $${\left|\left(\cos\theta +i\sin\theta \frac{d}{dx}\right)f\left(x\right)\right|}^{2}\leq N\int_{-\sigma }^{\sigma }{\left|uK_{\theta }^{*}\left(u,x\right)\right|}^{2}du.$$

However,

(176) $$\int_{-\sigma }^{\sigma }{\left|uK_{\theta }^{*}\left(u,x\right)\right|}^{2}du=\frac{1}{2\pi \left|\sin\theta \right|}\int_{-\sigma }^{\sigma }{\left|u\right|}^{2}du=\frac{\sigma^{3}}{3\pi \left|\sin\theta \right|},$$

and therefore,

(177) $${\left|\left(\cos\theta +i\sin\theta \frac{d}{dx}\right)f\left(x\right)\right|}^{2}\leq N\frac{\sigma^{3}}{3\pi \left|\sin\theta \right|}.$$

## 9. Discrete Case

For the eigenvalue problem, we write

(178) $$A\;u_{n}\left(x\right)\;=\;a_{n}u_{n}\left(x\right),$$

where $a_{n}$ and $u_{n}\left(x\right)$ are the discrete eigenvalues and eigenfunctions, respectively. The normalization is

(179) $$\int \;u_{n}^{*}\left(x\right)\;u_{m}\left(x\right)\;dx\;=\;\delta_{nm}$$

(180) $$\;\sum_{n=0}^{\infty }\;u_{n}^{*}\left(x\right)u_{n}\left(x^{\prime }\right)=\;\delta \left(x-x^{\prime }\right).\;$$

Any function, f(x) , can be expanded as

(181) $$f\left(x\right)\;=\;\sum_{i=0}^{\infty }F_{i}u_{i}\left(x\right)$$

with

(182) $$F_{i}\;=\;\;\int_{-\infty }^{\infty }u_{i}^{*}\left(x\right)f\left(x\right)\;dx.$$

The set of $F_{i}$ is analogous to the continuous *F* defined in Section 1. However, in quantum mechanics, it is customary to use $c_{i}$ for the expansion coefficients, and therefore, we rewrite Equations (181) and (182) as

(183) $$f\left(x\right)\;=\;\sum_{i=0}^{\infty }c_{i}u_{i}\left(x\right)$$

with

(184) $$c_{i}\;=\;\;\int_{-\infty }^{\infty }u_{i}^{*}\left(x\right)f\left(x\right)\;dx.$$

We define a bandlimited function when

(185) $$c_{i}=0\;\mathrm{for}\;i<n\;\mathrm{and}\;i>m,$$

in which case,

(186) $$f\left(x\right)\;=\;\sum_{i=n}^{m}c_{i}u_{i}\left(x\right).$$

The normalization factor is

(187) $$N=\;\sum_{i=n}^{m}{\left|c_{i}\right|}^{2}=\;\int_{-\infty }^{\infty }{\left|f(x)\right|}^{2}\;dx.$$

Consider

(188) $$g\left(x\right)\;=\;\sum_{i=n}^{m}c_{i}h_{i}u_{i}\left(x\right),$$

where $h_{i}$ are arbitrary real numbers. Taking

(189) $${\left|g(x)\right|}^{2}\;={\left|\;\sum_{i=n}^{m}c_{i}h_{i}u_{i}\left(x\right)\right|}^{2}$$

and using the Schwarz inequality for the discrete case

(190) $$\sum_{i=n}^{m}{\left|a_{i}\right|}^{2}\times \sum_{i=n}^{m}{\left|b_{i}\right|}^{2}\geq {\left|\;\sum_{i=n}^{m}a_{i}b_{i}^{*}\right|}^{2}$$

with

(191) $$\begin{array}{cc} \lambda_{i} & =c_{i} \end{array}$$

(192) $$\begin{array}{cc} b_{i} & =h_{i}u_{i}\left(x\right), \end{array}$$

we obtain

(193) $$\;\sum_{i=n}^{m}{\left|c_{i}\right|}^{2}\times \sum_{i=n}^{m}{\left|h_{i}u_{i}\left(x\right)\right|}^{2}\geq {\left|\;\sum_{i=n}^{m}c_{i}h_{i}^{*}u_{i}^{*}\left(x\right)\right|}^{2},$$

which gives

(194) $$N\;\sum_{i=n}^{m}{\left|h_{i}u_{i}\left(x\right)\right|}^{2}\geq {\left|\;\sum_{i=n}^{m}c_{i}h_{i}^{*}u_{i}^{*}\left(x\right)\right|}^{2}$$

or

(195) $${\left|g(x)\right|}^{2}\leq N\;\sum_{i=n}^{m}{\left|h_{i}u_{i}\left(x\right)\right|}^{2}.$$

### 9.1. $h_{i}$ = 1 Case

For

(196) $$h_{i}=1,$$

Equation (195) becomes

(197) $${\left|\;\sum_{i=n}^{m}c_{i}u_{i}\left(x\right)\right|}^{2}\leq N\sum_{i=n}^{m}{\left|u_{i}\left(x\right)\right|}^{2}$$

or

(198) $${\left|f(x)\right|}^{2}\leq N\sum_{i=n}^{m}{\left|u_{i}\left(x\right)\right|}^{2}.$$

### 9.2. Example: Hamiltonian

For a time-independent Hamiltonian, we write the eigenvalue problem as

(199) $$Hu_{n}\left(x\right)=E_{n}u_{n}\left(x\right),$$

where $E_{n}$ and $u_{n}$ are the discrete energy eigenvalues and eigenfunctions. The wave function may be expanded as

(200) $$\psi \left(x,t\right)=\sum_{i=0}^{\infty }c_{i}u_{i}\left(x\right)e^{iE_{i}t/\hbar }.$$

We define the bandlimited case by taking a finite range of terms

(201) $$\psi \left(x,t\right)=\sum_{i=k}^{n}c_{i}u_{i}\left(x\right)e^{iE_{i}t/\hbar }$$

with normalization

(202) $$N=\sum_{i=k}^{n}{\left|c_{i}\right|}^{2}.$$

Equation (198) gives

(203) $${\left|\psi (x,t)\right|}^{2}\leq N\sum_{i=k}^{n}{\left|u_{i}\left(x\right)\right|}^{2}.$$

Hence,

(204) $$\mathrm{Probability}\;\mathrm{of}\;\mathrm{position}\;={\left|\psi (x,t)\right|}^{2}\leq \frac{L}{2\hbar \pi }.$$

**Example:** As an example, consider the case where we have only two terms,

(205) $$\psi \left(x,t\right)=c_{k}u_{k}\left(x\right)e^{iE_{k}t/\hbar }+c_{n}u_{n}\left(x\right)e^{iE_{n}t/\hbar }.$$

The normalization factor is

(206) $$N={\left|c_{k}\right|}^{2}+{\left|c_{n}\right|}^{2}.$$

Equation (203) gives

(207) $${\left|c_{k}u_{k}\left(x\right)e^{iE_{k}t/\hbar }+c_{n}u_{n}\left(x\right)e^{iE_{n}t/\hbar }\right|}^{2}\leq \left({\left|c_{k}\right|}^{2}+{\left|c_{n}\right|}^{2}\right)\left({\left|u_{k}\left(x\right)\right|}^{2}+{\left|u_{n}\left(x\right)\right|}^{2}\right).$$

#### Example: Particle in a Box

For a particle in a box where

$$E_{n}=\frac{\pi^{2}\hbar^{2}}{2mL^{2}}n^{2}\;\;\;\;\;\;\;\;\;;\;\;\;\;\;\;\;\;u_{n}\left(x\right)=\sqrt{\frac{2}{L}}\sin\left(\frac{n\pi }{L}x\right)$$

(208) $$\psi \left(x,t\right)=c_{k}\sqrt{\frac{2}{L}}\sin\left(\frac{k\pi }{L}x\right)e^{iE_{k}t/\hbar }+c_{n}\sqrt{\frac{2}{L}}\sin\left(\frac{n\pi }{L}x\right)e^{iE_{n}t/\hbar },$$

Equation (207) gives that the probability is bounded by

(209) $${\left|\psi (x,t)\right|}^{2}\leq \left({\left|c_{k}\right|}^{2}+{\left|c_{n}\right|}^{2}\right)\left(\frac{2}{L}\sin^{2}\left(\frac{k\pi }{L}x\right)+\frac{2}{L}\sin^{2}\left(\frac{n\pi }{L}x\right)\right)$$

or

(210) $$\begin{array}{cc} & {\left|c_{k}\sqrt{\frac{2}{L}}\sin\left(\frac{k\pi }{L}x\right)e^{iE_{k}t/\hbar }+c_{n}\sqrt{\frac{2}{L}}\sin\left(\frac{n\pi }{L}x\right)e^{iE_{n}t/\hbar }\right|}^{2} \end{array}$$

(211) $$\begin{array}{cc} & \leq \left({\left|c_{k}\right|}^{2}+{\left|c_{n}\right|}^{2}\right)\left(\frac{2}{L}\sin^{2}\left(\frac{k\pi }{L}x\right)+\frac{2}{L}\sin^{2}\left(\frac{n\pi }{L}x\right)\right). \end{array}$$

## 10. The Dual Case

We consider that the quantum mechanical case of a particle is confined to a box of length *L*. The corresponding momentum wave function is

(212) $$\varphi \left(p\right)=\frac{1}{\sqrt{2\pi \hbar }}\int_{-L/2}^{L/2}\psi \left(x\right)e^{-ixp/\hbar }\;dx.$$

We obtain bounds on the momentum wave function. We assume that the wave function is normalized to one

(213) $$\int_{-L/2}^{L/2}{\left|\psi (x)\right|}^{2}dx=\int_{-\infty }^{\infty }{\left|\varphi (p)\right|}^{2}dp=1.$$

Consider now the momentum wave function of g(p)=ψ(x)H(x) where H(x) is an arbitrary real function

(214) $$g\left(p\right)=\frac{1}{\sqrt{2\pi \hbar }}\int_{-L/2}^{L/2}\psi \left(x\right)H\left(x\right)e^{-ixp/\hbar }\;dx.$$

Taking the absolute square of both sides of Equation (214), we have

(215) $${\left|g(p)\right|}^{2}={\left|\frac{1}{\sqrt{2\pi \hbar }}\int_{-L/2}^{L/2}\psi \left(x\right)H\left(x\right)e^{-ixp/\hbar }\;dx\right|}^{2}.$$

Using the Schwarz inequality, we obtain

(216) $$\int_{-L/2}^{L/2}{\left|\psi (x)\right|}^{2}dx\times \int_{-L/2}^{L/2}{\left|H\left(x\right)e^{-ixp/\hbar }\right|}^{2}dx\geq {\left|\int_{-L/2}^{L/2}\psi \left(x\right)H\left(x\right)e^{-ixp/\hbar }\;dx\right|}^{2}$$

or

(217) $$\int_{-L/2}^{L/2}{\left|\psi (x)\right|}^{2}dx\times \int_{-L/2}^{L/2}{\left|H(x)\right|}^{2}dx\geq {\left|\int_{-L/2}^{L/2}\psi \left(x\right)H\left(x\right)e^{-ixp/\hbar }\;dx\right|}^{2}.$$

Assuming that ψ(x) is normalized to one, this reduces to

(218) $$\int_{-L/2}^{L/2}{\left|H(x)\right|}^{2}dx\geq {\left|\int_{-L/2}^{L/2}\psi \left(x\right)H\left(x\right)e^{-ixp/\hbar }\;dx\right|}^{2},$$

which we rewrite as

(219) $$\int_{-L/2}^{L/2}{\left|H(x)\right|}^{2}dx\geq 2\pi \hbar {\left|\int_{-L/2}^{L/2}\frac{1}{\sqrt{2\pi \hbar }}\psi \left(x\right)H\left(x\right)e^{-ixp/\hbar }\;dx\right|}^{2}.$$

Using the definition of g(p) as per Equation (214), we have

(220) $$\int_{-L/2}^{L/2}{\left|H(x)\right|}^{2}dx\geq 2\pi \hbar {\left|g(p)\right|}^{2}.$$

**Special cases**. For H(x)=1, we have

(221) $$\int_{-L/2}^{L/2}{\left|H(x)\right|}^{2}dx=L$$

and

(222) g(p)=φ(p).

Equation (220) then gives

(223) $$L\geq 2\pi \hbar {\left|\varphi (p)\right|}^{2}$$

or

(224) $$\left|\varphi (p)\right|\leq \sqrt{\frac{L}{2\pi \hbar }}.$$

For

(225) $$H\left(x\right)=x^{n}$$

(226) $$\int_{-L/2}^{L/2}x^{2n}dx=\frac{L^{2n+1}}{2^{2n}\left(2n+1\right)}$$

and also

(227) $$g\left(p\right)=\int_{-L/2}^{L/2}\psi \left(x\right)x^{n}e^{-ixp/\hbar }\;dx={\left(\frac{\hbar }{-i}\right)}^{n}\sqrt{2\pi \hbar }\;\frac{\partial^{n}}{\partial p^{n}}\varphi \left(p\right).$$

Therefore, Equation (220) now gives

(228) $$\frac{L^{2n+1}}{2^{2n}\left(2n+1\right)}\geq 2\pi \hbar^{2n+1}{\left|\frac{\partial^{n}}{\partial p^{n}}\varphi \left(p\right)\right|}^{2}$$

or

(229) $$\left|\frac{\partial^{n}}{\partial p^{n}}\varphi \left(p\right)\right|\leq \sqrt{\frac{1}{\pi (2n+1)}{\left(\frac{L}{2\hbar }\right)}^{2n+1}}.$$

For the momentum wave function (n=0) and its derivative (n=1) cases, we have the following bounds respectively.

The probability distribution of momentum is therefore bounded

(230) $$\mathrm{Probability}\;\mathrm{of}\;\mathrm{momentum}\;={\left|\varphi (p)\right|}^{2}\leq \frac{L}{2\hbar \pi },$$

and the first derivative is bounded by

(231) $$\left|\frac{\partial }{\partial p}\varphi \left(p\right)\right|\leq \sqrt{\frac{1}{3\pi }{\left(\frac{L}{2\hbar }\right)}^{3}}.$$

### 10.1. Example: Particle in a Box

Consider the following normalized wave function:

(232) $$\psi \left(x\right)=\sqrt{\frac{2}{L}}\;\cos\frac{\pi x}{L}\;\left|x\right|\leq L/2.$$

Using Equation (224), we have the constraint

(233) $$\mathrm{Probability}\;\mathrm{of}\;\mathrm{momentum}\;={\left|\varphi (p)\right|}^{2}\leq \frac{L}{2\pi \hbar }.$$

The momentum wave function corresponding to Equation (232) can be calculated exactly,

(234) $$\begin{array}{cc} \varphi (p) & =\frac{1}{\sqrt{2\pi \hbar }}\int_{-L/2}^{L/2}\sqrt{\frac{2}{L}}\cos\frac{\pi x}{L}e^{-ixp/\hbar }\;dx \end{array}$$

(235) $$\begin{array}{cc} & =2\sqrt{\frac{\pi L}{\hbar }}\;\frac{\;\cos\;\left(\frac{pL}{2\hbar }\right)}{\pi^{2}-{\left(\frac{pL}{\hbar }\right)}^{2}}, \end{array}$$

and hence,

(236) $$\mathrm{Probability}\;\mathrm{of}\;\mathrm{momentum}\;=\frac{4\pi L}{\hbar }\;{\left[\frac{\;\cos\;\left(\frac{pL}{2\hbar }\right)}{\pi^{2}-{\left(\frac{pL}{\hbar }\right)}^{2}}\right]}^{2}\leq \frac{L}{2\pi \hbar }.$$

### 10.2. Example: Truncated Gaussian in Box

We consider the normalized truncated Gaussian wave function

(237) $$\psi \left(x\right)=\;\frac{1}{\pi^{1/4}\sqrt{L\;\mathrm{erf}\;\left(1/2\right)}}\exp\;\left(-\frac{x^{2}}{2L^{2}}+ixp_{0}/\hbar \right)\;\left|x\right|\leq L/2.$$

The momentum wave function is defined by

(238) $$c\left(p\right)=\frac{1}{\sqrt{2\pi \hbar }}\int_{-L/2}^{L/2}\psi \left(x\right)\;e^{-ipx/\hbar }\;dx,$$

which is constrained by

(239) $$\mathrm{Probability}\;\mathrm{distribution}\;\mathrm{of}\;\mathrm{momentum}:\;{\left|\varphi (p)\right|}^{2}\leq \frac{L}{2\pi \hbar }.$$

Note that the momentum wave function does not have to be calculated to obtain Equation (239). In this case, it can be

(240) φ(p)=Lπ1/4ℏ erf(1/2)exp −L2(p−p0)22ℏ2Re erf 122+i L(p−p0)2 ℏ,

and the exact probability distribution of momentum is

(241) |φ(p)|2=Lπ ℏ erf(1/2)exp −L2(p−p0)2ℏ2Re2 erf 122+i L(p−p0)2 ℏ2.

### 10.3. Quantum Position Bump Function

Bump functions are a function defined on a finite interval where all of its derivatives at the endpoints exist [24]. We consider here the following normalized bump function:

(242) $$\psi \left(x\right)=\frac{1}{C}\exp\;\left(-\frac{L^{2}}{L^{2}-4x^{2}}\right)\;\left|x\right|<L/2$$

(243) $$\psi \left(x\right)=\frac{1}{C}\exp\;\left(-\frac{1}{1-4{\left(x/L\right)}^{2}}\right)\;\left|x\right|<L/2,$$

where

(244) $$C=L\int_{-1}^{1}\exp\;\left(-\frac{2}{1-t^{2}}\right)dt$$

(245) $$\mathrm{Probability}\;\mathrm{distribution}\;\mathrm{of}\;\mathrm{momentum}:\;{\left|\varphi (p)\right|}^{2}\leq \frac{L}{2\pi \hbar }.$$

The momentum wave function cannot be expressed in a simple closed form. In [25], the asymptotic behavior is considered.

## 11. Conclusions

We have generalized the concept of bandlimited functions to representations other than the Fourier representation. In general terms, we have shown that if a function is bounded in one domain, then the inequalities we have derived place bounds on the function and its derivatives in the transformed domain. In the quantum mechanical case, these inequalities apply to probability distributions of position and momentum, as well as to derivatives of the wave functions. In particular, for a particle constrained to a finite interval, for example, a particle in a box with an arbitrary wave function, the inequalities show that the momentum probability distribution has an upper bound. While these inequalities provide upper bounds, they are not necessarily the best possible ones.

An important issue concerns the uncertainty principle and bandlimited functions. This is a historical question that goes back to the beginnings of the concept of bandlimited frequency functions. The classical paper on this subject is by Slepian [26]. We are currently investigating this issue in connection with the generalized uncertainty principle, that is, the uncertainty principle for two noncommuting operators.

Of particular interest in the quantum mechanical case are bounds on phase and amplitude. While we have briefly mentioned this issue in Section 4 in connection with instantaneous spatial frequency, we are currently investigating how these approximations behave in relation to our recent work on the relative importance of phase and amplitude [27].

Issues that are currently being investigated include extensions to the three-dimensional case and, more generally, to multidimensional wave functions. Extensions to wave functions with spin, as well as to wave functions with angular momentum, are also under study.

The study of bandlimited wave functions is particularly interesting in quantum mechanics. Generally speaking, for such wave functions, derivatives at the endpoints do not exist. We have introduced, from the field of signal processing, quantum bump wave functions, which are bandlimited functions for which all derivatives do exist at the endpoints. To the best of the authors’ knowledge, these types of wave functions and their possible applications have not been previously studied in quantum mechanics.

One of the referees suggested that the methods developed be applied to the fractional Fourier transform. We have done so briefly in Section 8, but this topic requires considerable further investigation. In addition, one of the referees asked whether the methods developed apply to the Wigner distribution and, by implication, to the general class of quasi-distributions. This is a very interesting question and is currently being investigated.

A natural question is whether the methods can be extended to quantum averages in order to obtain bounds on expectation values. This can be achieved by considering

(246) $$g\left(x\right)=\frac{1}{\sqrt{2\pi \hbar }}\int_{-L/2}^{L/2}\varphi \left(p\right)\;H\left(p\right)\;G\left(p\right)\;e^{ipx/\hbar }\;dp$$

instead of Equation (86), where we have added an additional arbitrary function G(p). Defining the expectation value of ${\left|G\left(p\right)\right|}^{2}$ by

(247) $${\langle |G\left(p\right)|}^{2}\rangle =\int_{-L/2}^{L/2}{\left|G\left(p\right)\right|}^{2}\;{\left|\varphi \left(p\right)\right|}^{2}\;dp,$$

we have been able to obtain constraints on $\langle |G\left(p\right){|}^{2}\rangle$. Such constraints are of particular interest in quantum chemistry. In addition, these methods can be applied to reduced density matrices as defined in quantum chemistry, thereby placing bounds on one and two body properties.

## Data Availability

The original contributions presented in this study are included in the article. Further inquiries can be directed to the corresponding author.
